# The impact of advertising patient and public involvement on trial recruitment: embedded cluster randomised recruitment trial

**DOI:** 10.1186/s13063-016-1718-1

**Published:** 2016-12-08

**Authors:** Adwoa Hughes-Morley, Mark Hann, Claire Fraser, Oonagh Meade, Karina Lovell, Bridget Young, Chris Roberts, Lindsey Cree, Donna More, Neil O’Leary, Patrick Callaghan, Waquas Waheed, Peter Bower

**Affiliations:** 1 York Trials Unit, Department of Health Sciences, University of York, York, YO10 5DD UK; 2 Division of Population Health, Health Services Research and Primary Care, University of Manchester, Williamson Building, Manchester, M13 9PL UK; 3 Division of Nursing, Midwifery and Social Work, University of Manchester, Jean McFarlane Building, Manchester, M13 9PL UK; 4 School of Health Sciences, Queen’s Medical Centre, The University of Nottingham, Nottingham, NG7 2HA UK; 5 MRC North West Hub for Trials Methodology Research, Institute of Psychology, Health and Society, University of Liverpool, Liverpool, UK; 6 HRB Clinical Research Facility, National University of Ireland Galway, Galway, Republic of Ireland; 7 School of Health Sciences and Institute of Mental Health, The University of Nottingham, Jubilee Campus, Nottingham, NG7 2TU UK; 8 MRC North West Hub for Trials Methodology Research, Manchester Academic Health Science Centre, University of Manchester, Williamson Building, Manchester, M13 9PT UK

**Keywords:** Recruitment, Patient and public involvement, Research methodology, Randomised controlled trial, Service user involvement, Study within a trial, *Mesh*: embedded trial

## Abstract

**Background:**

Patient and public involvement in research (PPIR) may improve trial recruitment rates, but it is unclear how. Where trials use PPIR to improve design and conduct, many do not communicate this clearly to potential participants. Better communication of PPIR might encourage patient enrolment, as trials may be perceived as more socially valid, relevant and trustworthy. We aimed to evaluate the impact on recruitment of directly advertising PPIR to potential trial participants.

**Methods:**

This is a cluster trial, embedded within a host trial (‘EQUIP’) recruiting service users diagnosed with severe mental illness. The intervention was informed by a systematic review, a qualitative study, social comparison theory and a stakeholder workshop including service users and carers. Adopting Participatory Design approaches, we co-designed the recruitment intervention with PPIR partners using a leaflet to advertise the PPIR in EQUIP and sent potential participants invitations with the leaflet (intervention group) or not (control group). Primary outcome was the proportion of patients enrolled in EQUIP. Secondary outcomes included the proportions of patients who positively responded to the trial invitation.

**Results:**

Thirty-four community mental health teams were randomised and 8182 service users invited. For the primary outcome, 4% of patients in the PPIR group were enrolled versus 5.3% of the control group. The intervention was not effective for improving recruitment rates (adjusted OR = 0.75, 95% CI = 0.53 to 1.07, *p* = 0.113). For the secondary outcome of positive response, the intervention was not effective, with 7.3% of potential participants in the intervention group responding positively versus 7.9% of the control group (adjusted OR = 0.74, 95% CI = 0.53 to 1.04, *p* = 0.082). We did not find a positive impact of directly advertising PPIR on any other outcomes.

**Conclusion:**

To our knowledge, this is the largest ever embedded trial to evaluate a recruitment or PPIR intervention. Advertising PPIR did not improve enrolment rates or any other outcome. It is possible that rather than advertising PPIR being the means to improve recruitment, PPIR may have an alternative impact on trials by making them more attractive, acceptable and patient-centred. We discuss potential reasons for our findings and implications for recruitment practice and research.

**Trial registration numbers:**

ISRCTN, ISRCTN16488358. Registered on 14 May 2014.

Study Within A Trial, SWAT-26. Registered on 21 January 2016.

**Electronic supplementary material:**

The online version of this article (doi:10.1186/s13063-016-1718-1) contains supplementary material, which is available to authorized users.

## Background

Randomised controlled trials are the ‘gold standard’ for evaluating treatments, yet recruitment into trials remains a great challenge, with approximately 45% of publicly funded and 80% of industry-funded trials failing to meet their recruitment targets [1, 2]. Mental health disorders are the leading cause of disability among adults worldwide [3]; however, trials enrolling patients with mental health problems experience even greater recruitment challenges [4–7]. These challenges stem from various sources including stigma [8] and issues related to the diagnosis adversely impacting on the patient’s ability and motivation to participate in research [9]. Inability to recruit into a trial adversely impacts trials by reducing the total sample size (which limits internal validity) and the proportion of eligible participants who are recruited (which limits external validity).

Thus there is a need to develop and test interventions to improve recruitment. One method is to ‘embed’ trials of recruitment interventions in ongoing trials; however, such trials are rare. Systematic reviews of trial recruitment interventions have highlighted the need for more embedded recruitment trials [10, 11]. Recent initiatives have also increasingly called for the development and evaluation of interventions for recruiting and retaining participants in trials [11–16].

We have developed methodological, logistical and reporting frameworks for embedded recruitment trials [12, 17] and assessed their feasibility using interventions such as an improved Participant Information Sheet and a multimedia decision aid [18, 19]. The eventual aim is to make delivery of embedded recruitment trials a routine activity, to assist the rapid development of recruitment to meet health and policy goals [20].

Patient and public involvement in research (PPIR), also known, among other terms, as ‘user involvement’, is research being carried out ‘with’ or ‘by’ patients and/or members of the public rather than ‘to’, ‘about’ or ‘for’ them [21]. This definition of PPIR is broad and involves patients, all groups who represent patients, as well as members of the public taking roles in the development, conduct and governance of research [22–24]. PPIR is thought to be crucial because it produces ‘better’ patient-focussed research by offering unique, invaluable insights into its prioritization, design, implementation and evaluation, making trials more effective and credible [25, 26]. PPIR is well-established as public policy in the United Kingdom (UK) and other developed countries and is increasingly mandated for publicly funded trials [27–30]. However, quantitative evidence around its impact is sparse, and that which exists is of poor quality and lacking in rigour [31]. There is a need to assess the effectiveness, cost-effectiveness and ethical impacts of PPIR through high-quality methodological research [31–37].

We recently reported a systematic review and meta-synthesis of factors affecting the recruitment of participants into depression trials [38] to help us to develop and evaluate an intervention for recruiting participants into mental health trials, using the Medical Research Council (MRC) complex interventions framework [39]. We developed a conceptual framework, which highlighted that the decision by patients to enrol as subjects in trials involves a difficult deliberation involving ‘risk’ [38]. This includes potential risks of stigma, of ‘losing out’ by being randomised to the ‘wrong’ intervention arm, or of encountering adverse effects of trial involvement, against potential rewards such as a personal need to access treatment and support. Outside of the mental health context, perceptions of risk have also been shown to impact on patients’ decision to enrol in trials [40–44]. We have also undertaken a qualitative study with patients who declined to participate in a trial, which highlighted the need to research the presentation and provision of accurate and effective trial information in which patients and the public play a seminal role [45].

There is some emerging observational evidence that mental health trials with more PPIR are associated with an increased likelihood of achieving their recruitment targets [46], although studies in other clinical settings have had variable outcomes [47]. PPIR may have a role in reducing patient perception of risk in trials and, as a consequence, may increase trial enrolment. Patients may perceive trials with PPIR to be improved methodologically or ethically, or to be more relevant and, therefore, more likely to influence practice in ways that are important to them and other patients [25, 26, 48]. Additionally, the concept of ‘social validation’ suggests that people may be more willing to comply with a request to enrol in a trial if they believe that others are already engaged in a trial, as people tend to compare and base their beliefs, attitudes and actions on similar others [49–51]. A survey of public attitudes to research suggests that PPIR may increase confidence and trust in a trial, if potential participants are reassured that other patients have advised its design [52, 53]. The authors concluded that: *‘if health researchers communicate the fact that patients and the public have been involved in the design of their research when approaching potential study participants, it might help to boost recruitment’* [52, 53]. However, to achieve these effects, it is necessary that PPIR is communicated to patients, but this does not always seem to be the case as researchers tend not to routinely advertise PPIR [54, 55]. We aimed to test this hypothesis about the effects of PPIR on recruitment using a rigorous evaluation. In this paper, we describe the development and evaluation of an intervention directly advertising PPIR in a mental health trial to potential participants.

### Objectives

Our objectives were to work with PPIR stakeholders to develop an intervention directly advertising PPIR in the design and conduct of a host trial, the ‘Enhancing the Quality of User Involved care Planning in mental health services’ (‘EQUIP’) trial, which was recruiting people with a diagnosis of severe mental illness; and to evaluate its effectiveness on recruitment by undertaking a randomised controlled trial, embedded in the EQUIP host trial.

## Methods

We report the development of the intervention in line with the Criteria for Reporting the Development and Evaluation of Complex Interventions (CReDECI 2) [56] and its evaluation in line with the ‘guidelines for reporting embedded recruitment trials’ [17].

### Trial design: the EQUIP host trial

The EQUIP trial aimed to recruit 480 service users with diagnoses of severe mental illness to evaluate the cost-effectiveness of a training intervention for mental health professionals in enhancing user involvement in care planning. EQUIP had significant high-quality PPIR and was awarded the 2014 UK Mental Health Research Network Prize for ‘Outstanding Carer Involvement’ [57].

EQUIP is a multicentre cluster randomised trial, where 36 community mental health teams in the Midlands and the North of England were randomly allocated to training or to usual care. In EQUIP mental health team clusters were ‘paired’ at the recruitment stage (based on size and geographic location) and randomised using minimisation in pairs to training or the control arm. Recruitment in the paired clusters then operated in parallel.

EQUIP used existing registers maintained by community mental health teams to recruit service users. Recruitment was undertaken by the UK Clinical Research Network Mental Health (CRN MH) clinical studies officers (CSOs) and research nurses, who, in conjunction with service users’ care coordinators, were responsible for accessing service user details, determining eligibility and mailing trial invitations. Invitations were posted to patients before randomisation of mental health teams occurred in EQUIP. To be eligible, patients had to be: aged 18 years or older; under the care of the community mental health team; have capacity to provide fully informed consent; and judged by their care coordinator to be well enough to complete study assessments. The research team did not have access to service users’ details until service users returned the ‘Consent to Contact’ Form. In the majority of mental health teams, potential participants who did not respond to the initial invitation letter were telephoned by a CSO or a member of their mental health team to determine whether they had received the trial invitation and whether they were interested in taking part. Recruitment and baseline assessment of participants within each cluster occurred within a 6-week period, before the training was delivered to the mental health clusters in the intervention arm. The EQUIP team aimed to recruit a minimum of 10 participants per cluster (no upper limit was specified). Details of the EQUIP trial design have been reported elsewhere [58].

### Trial design: the embedded recruitment trial

Recruitment into the embedded trial occurred over an 18-month period (June 2014 to December 2015) until recruitment into the host trial ceased. A patient-level randomised controlled trial would have been the most efficient design for the embedded recruitment trial; however, this was not practical as it was logistically burdensome for the EQUIP host trial team to administer. We therefore adopted a cluster randomised design for the recruitment trial, using the same mental health team clusters as in the EQUIP host trial. This had two methodological implications. First, due to the relatively small numbers of clusters (*n* = 36), there was a possibility of imbalance between the patients in the two arms of the embedded trial. Second, there was also a potential risk to the validity of the host trial: if the PPIR recruitment intervention were successful there could be differences between arms in the numbers and types of patients enrolled into the host trial.

We therefore adopted a cross-factorial, embedded randomised controlled trial design with the EQUIP host trial intervention allocation, using pairwise allocation. In the embedded trial, the same cluster pairs as in the EQUIP host trial were presented for randomisation; however, we randomised *both* clusters to receive the PPIR intervention, or *both* to the control arm (as opposed to *one* cluster being assigned to the intervention arm, and the *other* to the control arm). The priority was to ensure the integrity of the host trial. Pairwise allocation guaranteed that we achieved balance of cluster allocations between intervention and control arms for both the EQUIP host trial and for the embedded recruitment trial; this allocation method also ensured the validity of both the host and embedded recruitment trial interventions.

**Fig. 1:**
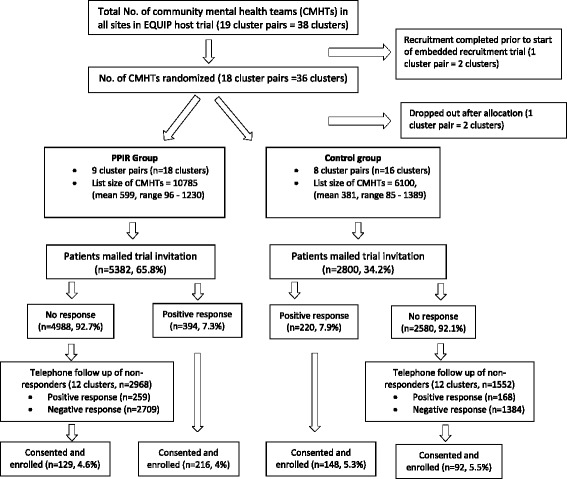
Flow diagram for the embedded recruitment trial. An overview of the flow of mental health teams and their patients in the embedded trial, based on the ‘guidelines for reporting embedded recruitment trials’, which adapts Consolidated Standards of Reporting Trials (CONSORT) for embedded recruitment trials [17]

Clusters were randomly allocated for their patients to be sent one of two interventions: the standard invitation (control group); or the PPIR intervention in addition to the standard invitation (intervention group). The PPIR intervention was sent in the same envelope as the EQUIP trial invitation, which also contained a cover letter, a Participant Information Sheet, a ‘Consent to Contact’ Form and stamped addressed envelope. The embedded recruitment trial thus measured the incremental benefit of being sent the recruitment intervention. Figure 1 outlines the recruitment flowchart for the embedded recruitment trial.

### Eligibility criteria for participants: embedded recruitment trial

The recruitment trial included all patients identified as potentially eligible for the EQUIP host trial: there were no additional inclusion or exclusion criteria.

### The recruitment intervention: the PPIR communication and its development

We developed a recruitment intervention communicating PPIR guided by the MRC complex interventions framework [39], informed by Participatory Design approaches with end users [59, 60]. As described earlier, the hypothesised mechanism was reducing the perception of risk in trial enrolment, informed by our prior systematic review [38] and qualitative study [45],‘social validation’, emerging from social comparison theory [49, 51] and survey evidence [52, 53]. We searched the latest Cochrane systematic reviews to determine frequently used recruitment and retention interventions [11, 61]. We reviewed the EQUIP host trial recruitment strategy and held discussions with the EQUIP team to determine a simple, systematic, feasible and acceptable method of delivering the PPIR intervention. Given that the recruitment occurred through mental health teams and patients were being approached to enter the host trial by postal invitations, we selected a leaflet format as the delivery mechanism to communicate PPIR. We then organised an expert workshop involving 27 key stakeholders including 10 service users with severe mental illness and two carers of people with severe mental illness, who were either EQUIP PPIR members or belonged to the EQUIP trial target population. Other stakeholders present were: five principal investigators/researchers with expertise in undertaking mental health trials; three patients with physical health problems; two researchers with expertise in PPIR; two mental health trial recruiters; two Research Ethics Board members and a consultant psychiatrist.

During this workshop, stakeholders endorsed the use of the of the leaflet format for advertising PPIR with the aim of improving recruitment. Working in small breakout groups (each group comprised of a mix of researchers and PPIR members), and then reconvening, stakeholders discussed and agreed seven ‘core principles’ for the leaflet advertising PPIR to potential trial participants (see Table 1).

**Table 1:** Core components of the patient and public involvement in research (PPIR) communication leaflet intervention

1. The intervention advertising PPIR was in a leaflet format
2. The leaflet was in a booklet style
3. The leaflet was written in plain language, with an informal, conversational style
4. The leaflet included photographs of the PPIR patients and carers, who in their own voice describe *how* they were involved in the trial and what their impact has been
5. The leaflet included photographs of the research team
6. The leaflet aimed to show that PPIR was taken seriously and was not tokenistic, and aimed to provide an honest account of PPIR
7. The leaflet aimed to be eye catching: bold, bright print, large font, colourful

In line with the principles of Participatory Design, participants were asked to design their ideal PPIR leaflet according to the ‘core principles’ in four breakout groups using appropriate materials. Each of the four groups presented their prototype leaflets to the wider group, including the key elements of the design. Members then voted for which of the four leaflets they thought was best overall for attracting potential participants. The top-rated leaflet contained similar elements to the other leaflets, including: making a clear and direct appeal for potential participants to join the trial; positive photographs of people with mental health problems which avoided the typical media image of people holding their heads in their hands, which members discussed as stigmatising [62]; highlighting benefits to future patients and convenience; the option to withdraw from the trial without giving a reason; and approval by an independent Research Ethics Committee.

Two of the PPIR members of EQUIP who were present at the workshop (LC and DM) – one a carer and the other a service user – volunteered to be photographed and featured in the EQUIP PPIR leaflet. Both PPIR members had active and ongoing involvement in EQUIP, one as a co-applicant and a member of the Trial Management Team; and both as part of the training team who delivered the user-involvement training intervention to the host trial intervention clusters. We worked closely with LC and DM to develop a bespoke leaflet for the EQUIP host trial, in line with the ‘core principles’ and taking into account key elements from the four leaflets created during the workshop. Once the initial version was developed, we asked for contributions from the EQUIP host trial researchers (chiefly to check for accuracy); their input did not change the content or format of the leaflet. The leaflet was then sent to a professional graphic designer in a company with significant expertise in designing patient communication materials (www.makingsense.co.uk). The design brief highlighted the agreed ‘core principles’ (Table 1) and related solely to the visual presentation of the leaflet and not the content. Two versions of the leaflet were initially designed and presented to the EQUIP team and PPIR members, who voted on their preferred design. Voting gave priority to PPIR members, who also provided comments in three rounds of iterations before the final design was agreed. These comments related to the colours and visual presentation, and the content did not change. Table 2 outlines the presentation and content of the final leaflet, which is also attached as Additional file 1.

**Table 2:** Content and layout of the finalised patient and public involvement in research (PPIR) leaflet

Presentational elements	Content
• Four-page booklet format • Photographs of the EQUIP trial team together with PPIR members on the front and back pages • Written in plain language; informal, conversational style • Contained several photographs of the PPIR members, including one of them designing the leaflet • Quotations written by PPIR members • Use of large font sizes and bright colours	• Front and back pages advertised award of ‘outstanding carer involvement’ to EQUIP • Front page stated that ‘real patients and carers’ had informed in the design of the study, and asked patients to consider taking part in EQUIP • Middle pages of the leaflet contained photographs of PPIR members • Quotations by PPIR members described why they thought the study was important • A section highlighting issues felt to be important to patients including: helping future patients, convenience, confidentiality, approval by a Research Ethics Committee • Quotation by EQUIP chief investigator about close working with PPIR members • Contained contact details of the study team

#### Outcome measures

In the EQUIP host trial, CSOs or mental health teams telephoned patients who did not initially respond to the postal invitation in poor recruiting clusters. There is evidence that telephone follow-up prompting of patients who do not respond to invitations to participate in trials significantly increases recruitment [11]. The host trial recruiters undertook telephone follow-ups as, and when, necessary which meant that not all clusters had the telephone follow-ups.

Our pre-planned primary outcome was, therefore, chosen to assess the effect of the PPIR leaflet, without potential contamination of the telephone follow-ups. The primary outcome for our embedded recruitment trial was the proportion of participants in each group who were *consented and enrolled* into the EQUIP host trial after responding to the postal invitation (i.e. the proportion of participants who responded and enrolled without the need for a telephone follow-up reminder).

The secondary outcomes were:

1. The proportion of patients in each group who *positively responded* without the need for a telephone follow-up reminder (note this differs from the number actually consented and enrolled, due to for instance, the EQUIP trial exclusion criteria)
2. The total proportions of patients in each group who were *consented and enrolled*, including telephone follow-up of initial nonresponders
3. The numbers of clusters in each group needing to conduct telephone follow-ups due to low postal response. This outcome takes into account the potential resource implications of a mental health clinician or a trial recruiter telephoning patients who do not respond to the trial invitation

### Sample size calculation

The sample size calculations for the EQUIP trial have been published in the original protocol [58].

As is usual with a trial embedded within a host trial, we did not undertake a formal power calculation to determine the sample size [15], since the sample size was constrained by the number of mental health teams and patients being approached in the EQUIP host trial. Our sample size was the total number of service users invited to participate in EQUIP from the 34 available clusters at the time of implementing the embedded trial, which was 8182 potential participants. We did not undertake a post-hoc power calculation as this is arguably a futile exercise, since the power of a trial is expressed in the confidence interval generated from the outcome analysis [63] (see ‘Results’ section).

### Randomisation

Randomisation was undertaken by the host trial statisticians (NO’L and CR), who were independent from the delivery of the trial interventions for both the host and embedded recruitment trials. Randomisation in the EQUIP host trial was stratified by cluster pairing, the site/region of each cluster and the caseload size of each cluster. For the recruitment trial, we used the same cluster pairing as the host trial and allocated each cluster pair by block-randomisation with permuted block sizes of 2, 4 and 6, using a computerised randomisation programme. Service users did not know that they were part of a trial of a recruitment intervention so were blind to the study hypothesis. CSOs and research nurses undertaking trial recruitment and mental health team clusters were also blind to the group to which clusters were allocated.

### Statistical methods

We obtained baseline data on cluster size (patient list size), deprivation, care quality rating and patient satisfaction with clinical care. Deprivation used the Index of Multiple Deprivation (IMD) rank averaged across Lower-layer Super Output Areas for each cluster’s Clinical Commissioning Group [64]. Care quality and patient satisfaction data were obtained at the cluster level from the Care Quality Commission which is the independent regulator of health and social care in England [65]. Patient satisfaction focussed on the experiences of service users who receive care and treatment within the mental health teams. Preliminary graphical and tabular examination of the data explored baseline comparability of trial arms and representativeness of the sample in terms of the clusters and the overall eligible population.

Data analysis used generalised linear mixed models [66] to estimate the effect of the recruitment intervention. As the unit of randomisation was the cluster pair, we fitted a three-level, random effects logistic model which pertained to the individual patient, clustered within mental health teams, and clustered within paired mental health teams. We adjusted for mental health team cluster size, levels of deprivation and care quality rating (we did not include patient satisfaction with clinical care in the model due to incomplete data). We present the marginal mean difference in proportions, as well as odds ratios (ORs), to assist with interpretation. Standard errors and confidence intervals for cluster marginal effects were calculated using the Delta Method. Given that the EQUIP randomisation occurred after the embedded trial randomisation, there was no plausible causal effect of the EQUIP intervention on recruitment so we did not test for an interaction between the EQUIP intervention and the recruitment intervention. Fisher’s exact test was used to test for association between recruitment trial arm and the need for telephone follow-up. Analyses used the intention-to-treat principle and were conducted using Stata, version 14 (Stata Corp., College Station, TX, USA).

## Results

Thirty-eight community mental health team clusters were recruited and randomised. One cluster pair (two clusters) could not be included as the EQUIP recruitment started before the embedded trial could begin. Another cluster pair withdrew from the EQUIP trial after randomisation, but prior to the mailing of invitation letters to their patients, and so are not included in the analysis. Eight thousand one hundred and eighty-two patients in 34 clusters were sent the standard EQUIP trial invitation letter or the addition of the PPIR intervention – see Fig. 1, flow diagram for the embedded recruitment trial. Table 2 outlines the characteristics of the mental health clusters and patients. Comparison of cluster baseline characteristics showed that clusters in the intervention arm were larger (544 mean patient list size versus 323); located in more deprived areas (IMD quintile median 1.5 versus 2.5); and had fewer mental health team clusters rated as ‘good’ for care quality (11.1% versus 18.8%). Patients in the intervention and control arms were broadly similar in age and gender distribution.

### Primary outcome

For the primary outcome of the proportions consented and enrolled into the EQUIP trial, 4% of patients sent the PPIR communication were enrolled compared with 5.3% of the control group (Table 3). Mixed-effects logistic regression showed that the recruitment intervention was not effective for improving recruitment rates [OR = 0.75, 95% CI = 0.53 to 1.07, *p* = 0.113]. The average marginal effect of the intervention on the probability of enrolment was −0.0123 [95% CI = −0.0282 to 0.0036].

**Table 3:** Baseline information for mental health cluster teams and patients, by allocation

Mental health team cluster Baseline factors	PPIR group	Control group
List size, mean (SD)	544 (273)	323 (191.6)
IMD quintile, median (range) [1 = most deprived; 5 = least deprived]	1.5 (1–4)	2.5 (1–5)
Care Quality Commission rating		
Good, *n* (%)	2 (11.1%)	3 (18.8%)
Requires improvement, *n* (%)	8 (44.4%)	6 (37.5%)
Rating suspended, *n* (%)	8 (44.4%)	4 (25%)
Not yet inspected, *n* (%)	0 (0%)	3 (18.8%)
Patient satisfaction with care^a^, mean (SD) [10 = highly satisfied]	6.6 (0.3)	6.9 (0.2)
Patients expressing interest^b^:		
Male, *n* (%)	151 (38.9%)	81 (36.8%)
Female, *n* (%)	237 (61.1%)	139 (63.2%)
Patients enrolled^b^:		
Male, *n* (%)	76 (36.2%)	49 (36%)
Female, *n* (%)	134 (63.8%)	87 (64%)
Mean age, years (SD)	48.5 (12.8)	45.5 (9.3)

*IMD* Index of Multiple Deprivation, *PPIR* patient and public involvement in research*, SD* standard deviation

^a^Patient satisfaction survey score data available for 32 clusters

^b^Baseline information only available for observed sample and not for entire cluster

### Secondary outcomes

1. Responding positively to the invitation, without telephone follow-up: there was no difference between the intervention and control groups, with 7.3% of potential participants sent the recruitment intervention responding positively, compared with 7.9% in the control group: adjusted OR = 0.74, 95% CI = 0.53 to 1.04, *p* = 0.082. The average marginal effect of the intervention on the probability of positive response was −0.0208 [95% CI = −0.0451 to 0.0035].
2. All positive response (including telephone follow-up): there was no difference between the intervention and control groups, with 9.2% of the intervention group responding positively, compared with 10.0% in the control group: adjusted OR = 0.74, 95% CI = 0.51 to 1.09, *p* = 0.125. The average marginal effect of the intervention on the probability of all positive response was −0.0343 [95% CI = −0.0795 to 0.0108].
3. Number of clusters requiring telephone follow-up of nonresponsive patients: this showed that there was no association between the recruitment trial arm and the need for a telephone reminder, with 66.7% in the PPIR group, compared with 75% of control group: Fisher’s exact test *p* value = 0.715.

#### Harms

We tested a two-tailed hypothesis, which accepted that sending the recruitment intervention to potential participants could cause benefit or loss to recruitment for the host trial. Patients not being recruited presents a loss to the host trial; however, for the patient, not being enrolled into the trial may not be harmful and may in fact be the best thing for them to make an informed decision that suits them without encountering the potential inconvenience or negative consequences of trial participation. The primary and secondary outcomes were designed to demonstrate any potential harms to the EQUIP host trial in terms of reduced enrolment in the intervention group. The results demonstrate that the recruitment intervention was ineffective for increasing enrolment rates for all outcomes measured. A second potential harm to the host trial was the potential differences in the numbers and types of patients enrolled into the host trial between the intervention and control groups. We sought to minimise this potential harm by adopting the cross-factorial design, and making baseline comparisons between the intervention and control groups. Baseline comparison of the intervention and control groups found no differences. We did not measure other potential harms, such as perceptions of increased pressure to participate in the intervention group.

## Discussion

### Summary of main findings

We undertook an embedded trial to evaluate the effectiveness on recruitment of directly advertising PPIR to potential trial participants. In this group of patients with severe mental health problems, the overall rates of response and participation were low, although this was in line with similar studies [18]. For our primary outcome, we found that being sent the intervention was not effective for improving recruitment rates. Our secondary outcomes found that directly advertising PPIR did not make a positive difference to any other outcomes.

### Strengths and limitations

To our knowledge, this multicentre trial involving 8182 patients is the largest-ever trial embedded in an ongoing trial to have been undertaken to evaluate the effectiveness of an intervention on trial recruitment [11] as well as the largest to evaluate the impact of patient and public involvement [31, 47]. Recruitment trials embedded within host trials are often plagued by the problem of small sample sizes as embedded trials are reliant upon the numbers of patients approached by the host trial. These numbers are not usually sufficient to show small but important differences in recruitment [11, 15].

The EQUIP host trial had award-winning high-quality PPIR. Additionally, the development of the recruitment intervention and its evaluation involved close collaboration with PPIR members. Both PPIR and recruitment are considered complex interventions [55, 67]. In our trial we used the MRC complex interventions framework to systematically develop a theory-informed recruitment intervention and evaluate it in a rigorous way, using real patients being approached to make a real decision about participation in an ongoing trial. With increasing calls and now guidance for measuring the impact of PPIR [68, 69], our work provides randomised evidence in a field that is very much lacking such evidence.

Cluster randomised designs are often used to evaluate the effectiveness of recruitment interventions [70–72], as they are often the most logistically feasible way to deliver recruitment interventions embedded in ongoing host trials. We recognise that cluster randomised trials can be susceptible to a range of methodological problems [73, 74]. Due to logistic and operational reasons, it was not possible to undertake a patient-level randomised trial, so we adopted a cluster randomised trial design, which was a design agreeable to the host trial team, and protected the host trial from potential biases introduced by the recruitment intervention such as differential recruitment and imbalance in the characteristics of patients recruited into the host trial as a consequence of the PPIR intervention. The outcome of the random allocation led to there being more and larger clusters in the intervention arm of the recruitment trial. However, this imbalance was a result of the random allocation and occurred by chance. This was a compromise and without this design we would not have been able to conduct the embedded recruitment trial, and we later adjusted for cluster size in the analyses. The randomisation of matched cluster pairs also has some potential problems, such as some pairs of clusters being more closely matched than others, so minimisation in this instance may have been a better option. However, again it was not feasible to undertake the minimisation because logistically, the least burdensome option for the host trial team was to use the same cluster pairs that they were using in the host trial.

There is an argument that the impact of involvement *within any particular project* is somewhat unpredictable, and that there is a need to provide details of context in accounts of PPIR [75]. Furthermore, there is also a need to understand how context and mechanism influence the impact of PPIR [75]. We did not have sufficient resources to undertake formal qualitative interviews to understand the mechanism of impact. However, we are currently undertaking two other embedded trials of this intervention directly advertising PPIR to potential trial participants to better understand the context and mechanism of impact. In one of these linked trials, we are undertaking user-testing of the PPIR recruitment intervention with patients and families to enable the revision and refining of the intervention to make it more appropriate to their context. We are also undertaking qualitative interviews with people who enter the host trial to explore their views of the PPIR intervention and determine its impact on their decision-making.

### Comparison with existing trial literature

Our findings contrast with a survey where 44% members of the British public responding to a hypothetical question indicated that they would be more likely to enrol in a trial if they found that patients had advised in its design [53]. The authors of this survey reported that very few people thought that PPIR would reduce their confidence in a trial. We found that patients actually invited to enter a real trial were no more likely to enrol when they were sent a leaflet about PPIR. Research investigating hypothetical and actual willingness to enrol in a trial found that only 20% of participants stating hypothetical willingness to enter a trial actually enrolled and that statements of hypothetical willingness to participate in future trials may overestimate true enrolment [76].

A systematic review to assesses the impact of PPIR on recruitment and retention in trials has found that while PPIR is consistently associated with improved retention, the evidence for impact on enrolment is variable and inconsistent [47]. A number of studies identified by this review found either no significant positive effect of PPIR on trial recruitment, or in one case involving the recruitment of African-Americans being recruited through three different sources, that the non-PPIR arm was more effective at improving recruitment [77]. Our present findings are, therefore, in line with the trial literature evaluating the impact of PPIR on recruitment.

### Explaining our findings and potential mechanisms of action

Beyond advertising PPIR intervention simply being ineffective for improving trial recruitment and response rates, there are a range of other possible reasons for our present findings. First, it is possible that people in the PPIR arm did not read the leaflet. The leaflet was sent by post in a large recruitment pack with several other documents. Those sent the recruitment pack may not have opened it, and those who did may not have read the PPIR recruitment leaflet. We were not able to determine how many people read the recruitment leaflet and our intention-to-treat analysis may have underestimated the effects of the active intervention components.

Second, it is also not clear whether those sent the leaflet, and who read it, understood the message in the leaflet and what PPIR meant for the trial that they were being asked to enrol into. Conversely, there is some research evidence indicating that patients receiving supplementary written information about a trial in the form of a booklet or leaflet have improved knowledge about the trial [78, 79]. It is possible that those who read the leaflet were more likely to make a more informed decision about not enrolling in the trial, which would have been a good decision for the patient, but a bad outcome for the trial. Unfortunately, in this population it was not possible to obtain estimates of the effect of the recruitment intervention for those who were randomised to receive the leaflet, who also actually read it, and how they interpreted the message.

Third, there are a range of mechanisms by which PPIR might influence recruitment, including on the trial design and trial conduct. Thus, the role of PPIR might lead to sensitive issues being handled better [80] or enhance trial quality and appropriateness, making them more effective [25, 35]. These mechanisms call into question the mechanism used in our trial, which is that advertising PPIR might improve recruitment. Additionally, the high-quality PPIR in EQUIP may have meant that the PPIR benefits may have been already optimised in EQUIP. The addition of the PPIR recruitment intervention may, therefore, have been irrelevant since the PPIR undertaken in EQUIP may have been sufficient to promote participant recruitment. However, the overall enrolment rates in EQUIP were low, with rates similar to other trials recruiting from similar populations [18, 81], so this does not suggest that the significant PPIR in EQUIP improved recruitment when compared with other trials. This contrasts with an observational study which found that studies that involved patients to a greater extent were more likely to have achieved recruitment targets [46].

Fourth, we developed our conceptual framework around the decision to enter trials using depression as the case exemplar, yet our recruitment intervention, informed by the conceptual framework, was evaluated in a population of patients with severe mental illness (who may or may not have had depression). Depression is the leading cause of disease burden worldwide [82, 83], and when we initiated our programme of work we anticipated that we would develop and then test the recruitment intervention using a depression trial. However, we found it impossible to recruit a host depression trial in order to evaluate the recruitment intervention, despite directly contacting 14 potential host trials registered on the National Institute for Health Research (NIHR) trials portfolio, seeking support from the Clinical Research Network Mental Health, which contacted trials on our behalf, and advertising for host trials via the UK Trial Managers’ Network. The main reason why a depression host trial was not forthcoming was due to a mismatch between the recruitment timelines of potential host trials and that of the embedded recruitment trial: the majority of host trials approached were either close to finishing participant recruitment, or were in the early phase of set-up, meaning that it was not possible to align host trial participant recruitment with the embedded recruitment trial. Two other depression trials reported that they already intended to advertise their PPIR activities to potential participants. This failure to recruit a depression trial may have impacted on the embedded trial outcomes as the intervention may not have been as relevant for the population in the EQUIP trial of people with severe mental illness. However, approximately 47% of the EQUIP population had comorbid depression. Additionally, mental health disorders in general have the strongest established history of PPIR in the UK [84–86], and there is some evidence that the use of PPIR is significantly associated with successful recruitment across a range of mental health trials, including severe mental illness, psychoses and depression [87, 88]. Furthermore, the Health Research Authority survey suggesting direct communication of PPIR to potential participants indicated that this approach could be used in all disease areas [52, 53]. We developed the PPIR intervention closely with the EQUIP trial and the use of the intervention was strongly endorsed by stakeholders.

Fifth, we used the concept of social validation to inform our recruitment intervention. The concepts of risk and social validation exist across all disease areas, however, not just depression. Social validation has also been used successfully as a trial recruitment intervention, with an embedded recruitment trial of text messages containing quotes from existing participants significantly increasing randomisations [89]. However, in our trial, social validation came from patients as *research partners*, rather than from patients as *trial participants*. This may have had an influence on our findings.

Finally, informal discussions of our findings with stakeholders suggested that the stigma associated with mental illness may have led to a negative impact of the PPIR intervention. Stigma, both towards others with mental health problems, as well as mental health stigma ‘internalised’ towards the person’s own self are well-documented and can deter people with mental health problems from seeking health care [90–92]. In our recruitment trial, stigma may have meant that awareness that EQUIP had significant PPIR from individuals with mental health problems may have made some people reluctant to enrol. Additionally, there may have been a perception of a lack of ‘professionalism’ in trial design and conduct, suggested by the significant involvement of patients and carers, as opposed to the trial being wholly conducted by ‘trained professional researchers’. Stigma and a perceived lack of professionalism may have combined to make some people disinclined to enrol in the trial. Other PPIR members and stakeholders involved in other trials suggested that the leaflet lacked representativeness and commented that the images of people in the leaflet were not representative of them, that: ‘*the people in this leaflet do not look like me*’. Diversity in representativeness of PPIR members has been discussed in the trial literature, and arguments have been made for the need to engage with PPIR representatives who reflect the diversity of the study population [93]. Due to resource constraints, we were unable to undertake qualitative interviews with the people sent the PPIR communication in order to explore and understand patient views of the intervention.

### Implications for recruitment practice, public policy and research

It is important to highlight here that while we found that directly communicating PPIR using a leaflet to potential trial participants was not effective for improving trial recruitment, this is not the same as PPIR being ineffective or harmful to trials *in general*. Our experience in undertaking this trial, and that of the EQUIP host trial, is that PPIR is very effective for developing interventions that can be delivered and evaluated in trials. However, we did not actually evaluate this, as the recruitment intervention was about direct communication of PPIR to potential participants. It is quite possible that rather than directly communicating PPIR to potential participants, what PPIR achieves in terms of making a trial and its interventions more attractive, acceptable and patient-centred is what is important in terms of its impact. More rigorous trials are needed to evaluate the impact of PPIR. Here, our findings point to a direction of focus for evaluating the impact of PPIR in trials, in informing the design and conduct of trials, but not as a means for direct recruitment. Policy-makers should be aware that PPIR is not a panacea and should fund more systematic evaluations of the impact of PPIR. Findings from this research will be sent to the authors of the Cochrane systematic review of interventions to improve recruitment to trials, for inclusion in future systematic reviews [11].

There is some evidence to suggest that PPIR may be effective for improving retention in trials [47]. Participants in EQUIP are currently in the follow-up phase. We aim to determine whether direct communication of PPIR improves retention in EQUIP. It is unclear to what extent different versions of this intervention might have had different impacts in different trial contexts and patient populations. For example, while the PPIR intervention was developed with PPIR partners, it was not user-tested with potential trial participants. There is some evidence that performance-based user-testing of trial information can identify strengths and weaknesses in trial information materials and make them fit for purpose [94, 95]. Here, the usability, acceptability and accessibility can be improved using semistructured interviews and iterative testing cycles [96]. A user-tested version of the intervention may have impacted on how potential participants responded. A user-tested version of the PPIR intervention is currently being evaluated in the Culturally-adapted Family Intervention (CaFI) study recruiting African-Caribbean people diagnosed with schizophrenia [97]; another version of the intervention is currently being evaluated in a study investigating early signs of dementia. Our broad aim is to aggregate the results across the different trials to obtain a more precise estimate of effect, as well as to explore the effectiveness of the intervention across different research contexts and patient populations.

Our trial highlights the potential benefits of process evaluation in embedded recruitment trials by adopting qualitative methods to explore patients’ use and views of recruitment interventions. This would make it necessary for trialists to obtain the necessary ethical permissions to approach people sent such recruitment materials to gain insights into the mechanisms and contexts of these interventions [98]. There are potential problems, as process evaluation would add significant costs to embedded trials and may add significant complexity to the process of embedding a trial, which might act as a barrier to adoption. In addition, however, our prior work with people who declined to enter a trial highlights that even those who declined to enter a trial reported that they do not mind being approached and, in addition, were happy to explore their trial participation decisions [45]. We are currently undertaking an additional qualitative study to explore the views of people who are sent a similar PPIR recruitment intervention as part of the trial embedded in the CaFI study [97].

## Conclusions

This embedded recruitment trial found no benefits of directly communicating PPIR on response, consent or enrolment rates. Further embedded trials of these materials are being conducted to explore how the impact of the intervention may vary by intervention type, trial context and patient population. A more comprehensive cohort of embedded trials of recruitment interventions across the trials portfolio could lead to a rapid development of the evidence base around recruitment to make trials more acceptable and accessible to patients.

## Additional file

Additional file 1: Recruitment intervention advertising patient and public involvement in research. A copy of the recruitment intervention which was mailed to potential trial participants. (PDF 2112 kb)

## Abbreviations

CaFI: Culturally-adapted Family Intervention
CMHT: Community Mental Health Team
CSO: Clinical studies officer
EQUIP: Enhancing the Quality of User Involved care Planning in mental health services
IMD: Index of Multiple Deprivation
ISRCTN: International Standard Randomised Controlled Trial Number
NHS: National Health Service
NIHR: National Institute for Health Research
NRES: National Research Ethics Service
OR: Odds ratio
PPIR: Patient and public involvement in research
UK: United Kingdom

## References

[1] Sully BG, Julious SA, Nicholl J (2013). A reinvestigation of recruitment to randomised, controlled, multicenter trials: a review of trials funded by two UK funding agencies. Trials.

[2] CenterWatch. State of the clinical trials industry 2009: a sourcebook of charts and statistics. Boston: CenterWatch; 2009.

[3] Whiteford HA, Ferrari AJ, Degenhardt L, Feigin V, Vos T (2015). The global burden of mental, neurological and substance use disorders: an analysis from the global burden of disease study 2010. PLoS One.

[4] Olsen K, Howel D, Barber R, Ford GA, Gallagher P, McAllister-Williams RH, Nilsson J, O’Brien J, Parker J, Thomas A (2015). Lessons from a pilot and feasibility randomised trial in depression (Blood pressure Rapid Intensive Lowering And Normal Treatment for Mood and cognition in persistent depression (BRILiANT mood study)). Pilot Feasibility Stud.

[5] Adams CE (2013). Many more reasons behind difficulties in recruiting patients to randomized controlled trials in psychiatry. Epidemiol Psychiatr Sci.

[6] Leeson VC, Tyrer P (2013). The advance of research governance in psychiatry: one step forward, two steps back. Epidemiol Psychiatr Sci.

[7] Bryant K, Wicks MN, Willis N (2014). Recruitment of older African American males for depression research: lessons learned. Arch Psychiatr Nurs.

[8] Woodall A, Morgan C, Sloan C, Howard L (2010). Barriers to participation in mental health research: are there specific gender, ethnicity and age related barriers?. BMC Psychiatry.

[9] Serretti A, Artioli P (2006). Ethical problems in pharmacogenetic studies of psychiatric disorders. Pharmacogenomics J.

[10] Watson JM, Torgerson DJ (2006). Increasing recruitment to randomised trials: a review of randomised controlled trials. BMC Med Res Methodol.

[11] Treweek S, Lockhart P, Pitkethly M, Cook JA, Kjeldstrom M, Johansen M, Taskila TK, Sullivan FM, Wilson S, Jackson C, Jones R, Mitchell ED (2013). Methods to improve recruitment to randomised controlled trials: Cochrane systematic review and meta-analysis. BMJ.

[12] Rick J, Graffy J, Knapp P, Small N, Collier D, Eldridge S, Kennedy A, Salisbury C, Treweek S, Torgerson D, Wallace P, Madurasinghe V, Hughes-Morley A, Bower P (2014). Systematic techniques for assisting recruitment to trials (START): study protocol for embedded, randomized controlled trials. Trials.

[13] Smith V, Clarke M, Devane D, Begley C, Shorter G, Maguire L (2013). SWAT 1: what effects do site visits by the principal investigator have on recruitment in a multicentre randomized trial?. J Evid Based Med.

[14] Treweek S (2013). Trial forge: a systematic approach to making trials more efficient. Trials.

[15] Adamson J, Hewitt CE, Torgerson DJ (2015). Producing better evidence on how to improve randomised controlled trials. BMJ.

[16] Anonymous. Education section—studies within a trial (SWAT). J Evid Based Med. 2015;8(3):165. 10.1111/j.1756-5391.2012.01169.x 23528122

[17] Madurasinghe VW, Eldridge S, on behalf of the MRC START Group and Gordon Forbes on behalf of the START Expert Consensus Group (2016). Guidelines for reporting embedded recruitment trials. Trials.

[18] Man MS, Rick J, Bower P (2015). Improving recruitment to a study of telehealth management for long-term conditions in primary care: two embedded, randomised controlled trials of optimised patient information materials. Trials.

[19] Bower P, Collier D, Eldridge S, Graffy J, Kennedy A, Knapp P, Hughes-Morley A, Rick J, Salisbury C, Small N (2013). A multimedia intervention to enhance recruitment to clinical trials in primary care and community settings: process of development and evaluation. Trials.

[20] National Institute for Health Research. Embedding health research: National Institute for Health Research Annual Report 2009/10. London: Department of Health; 2010. Crown Copyright.

[21] INVOLVE. Defining patient and public involvement. 2015.

[22] Stewart D, Wilson R, Selby P, Darbyshire J (2011). Patient and public involvement. Ann Oncol.

[23] Arnstein SR (1969). A ladder of citizen participation. J Am Inst Plann.

[24] Tritter JQ, McCallum A (2006). The snakes and ladders of user involvement: moving beyond Arnstein. Health Policy (New York).

[25] Entwistle VA, Renfrew MJ, Yearley S, Forrester J, Lamont T (1998). Lay perspectives: advantages for health research. BMJ.

[26] Staley K (2009). Exploring impact: public involvement in NHS, public health and social care research.

[27] Ives J, Damery S, Redwod S (2013). PPI, paradoxes and Plato: who’s sailing the ship?. J Med Ethics.

[28] Boote J, Telford R, Cooper C (2002). Consumer involvement in health research: a review and research agenda. Health Policy (New York).

[29] Gross D, Fogg ÃL (2001). Clinical trials in the 21st century: the case for participant-centered research. Res Nurs Health.

[30] Gagnon M-P, Desmartis M, Lepage-Savary D, Gagnon J, St-Pierre M, Rhainds M, Lemieux R, Gauvin F-P, Pollender H, Légaré F (2011). Introducing patients’ and the public's perspectives to health technology assessment: a systematic review of international experiences. Int J Technol Assess Health Care.

[31] Mockford C, Staniszewska S, Griffiths F, Herron-Marx S (2012). The impact of patient and public involvement on UK NHS health care: a systematic review. Int J Qual Heal Care.

[32] Evans D (2014). Patient and public involvement in research in the English NHS: a documentary analysis of the complex interplay of evidence and policy. Evid Policy A J Res Debate Pract.

[33] Forbat L, Hubbard G, Kearney N (2009). Patient and public involvement: models and muddles. J Clin Nurs.

[34] Brett J, Staniszewska S, Mockford C, Herron-Marx S, Hughes J, Tysall C, Suleman R (2014). A systematic review of the impact of patient and public involvement on service users, researchers and communities. Patient.

[35] Brett J, Staniszewska S, Mockford C, Herron-Marx S, Hughes J, Tysall C (2014). Mapping the impact of patient and public involvement on health and social care research: a systematic review. Heal Expect.

[36] Barber R, Boote J, Parry G (2012). Can the impact of public involvement on research be evaluated? A mixed methods study. Heal Expect.

[37] Staniszewska S, Adebajo A, Barber R, Beresford P, Brady L, Brett J (2011). Developing the evidence base of patient and public involvement in health and social care research: the case for measuring impact. Int J Consum Stud.

[38] Hughes-Morley A, Young B, Waheed W, Small N, Bower P (2015). Factors affecting recruitment into depression trials: systematic review, meta-synthesis and conceptual framework. J Affect Disord.

[39] Craig P, Dieppe P, Macintyre S, Michie S, Nazareth I, Petticrew M (2008). Developing and evaluating complex interventions: the new Medical Research Council guidance. BMJ.

[40] Reynolds WW, Nelson RM (2007). Risk perception and decision processes underlying informed consent to research participation. Soc Sci Med.

[41] Deakin CT, Alexander IE, Kerridge I (2009). Accepting risk in clinical research: is the gene therapy field becoming too risk-averse?. Mol Ther.

[42] Appelbaum PS, Lidz CW, Grisso T (2004). Therapeutic misconception in clinical research: frequency and risk factors. IRB.

[43] Snowdon C, Elbourne D, Garcia J (2007). Declining enrolment in a clinical trial and injurious misconceptions: is there a flipside to the therapeutic misconception?. Clin Ethics.

[44] Eborall HC, Stewart MC, Cunningham-Burley S, Price JF, Fowkes FGR (2011). Accrual and drop out in a primary prevention randomised controlled trial: qualitative study. Trials.

[45] Hughes-Morley A, Young B, Hempel R, Russell I, Waheed W, Bower P. What can we learn from trial decliners about improving recruitment? Qualitative study. Trials. 2016;17(494). 10.1186/s13063-016-1626-4 PMC5062905 27733181

[46] Ennis L, Wykes T (2013). Impact of patient involvement in mental health research: longitudinal study. Br J Psychiatry.

[47] Crocker J, Hughes-Morley A, Petit-Zeman S, Rees S (2015). Assessing the impact of patient and public involvement on recruitment and retention in clinical trials: a systematic review. Trials.

[48] Oliver SR, Rees RW, Clarke‐Jones L, Milne R, Oakley AR, Gabbay J, Stein K, Buchanan P, Gyte G (2008). A multidimensional conceptual framework for analysing public involvement in health services research. Heal Expect.

[49] Groves RM, Cialdini RB, Couper MP (1992). Understanding the decision to participate in a survey. Public Opin Q.

[50] Free CJ, Hoile E, Knight R, Robertson S, Devries KM (2011). Do messages of scarcity increase trial recruitment?. Contemp Clin Trials.

[51] Festinger L (1954). A theory of social comparison processes. Hum Relations.

[52] Hunn A. Survey of the general public: attitudes towards research. Ipsos MORI and NHS Health Research Authority; 2013. Available at: http://www.hra.nhs.uk/documents/2013/11/survey-general-public-attitudes-towards-health-research.pdf. Accessed 1 Dec 2013.

[53] Health Research Authority. Patient involvement increases public confidence in health research. 2013. [Online]. Available at: http://www.hra.nhs.uk/blog/news/2013/11/22/patient-involvement-increases-public-confidence-health-research/#sthash.rjPNrw7W.dpuf. Accessed 1 Dec 2013.

[54] Chambers R, O’Brien LM, Linnell S, Sharp S (2004). Why don’t health researchers report consumer involvement?. Qual Prim Care.

[55] Brett J, Staniszewska S, Mockford C, Seers K, Herron-Marx S, Bayliss H (2010). The PIRICOM Study: a systematic review of the conceptualisation, measurement, impact and outcomes of patients and public involvement in health and social care research.

[56] Möhler R, Kopke S, Meyer G (2015). Criteria for Reporting the Development and Evaluation of Complex Interventions in healthcare: revised guideline (CReDECI 2). Trials.

[57] National Institute for Health Research (2014). NIHR HS&DR study wins national award for involving service users and carers.

[58] Bower P, Roberts C, O’Leary N, Callaghan P, Bee P, Fraser C, Gibbons C, Olleveant N, Rogers A, Davies L, Drake R, Sanders C, Meade O, Grundy A, Walker L, Cree L, Berzins K, Brooks H, Beatty S, Cahoon P, Rolfe A, Lovell K (2015). A cluster randomised controlled trial and process evaluation of a training programme for mental health professionals to enhance user involvement in care planning in service users with severe mental health issues (EQUIP): study protocol for a randomised co. Trials.

[59] Sanders EB-N, Scrivener SAR, Ball LJ, Woodcock A (2000). Generative tools for co-designing. Collaborative design: proceedings of codesigning 2000.

[60] Sanders EB-N, Stappers PJ (2008). Co-creation and the new landscapes of design. CoDesign.

[61] Brueton VC, Tierney JF, Stenning S, Meredith S, Harding S, Nazareth I, Rait G. Strategies to improve retention in randomised trials: a Cochrane systematic review and meta-analysis. BMJ Open. 2014;4(2). 10.1136/bmjopen-2013-003821 PMC3918995 24496696

[62] Time to Change. Get the picture. 2016. [Online]. Available at: http://www.time-to-change.org.uk/getthepicture. Accessed 1 June 2016.

[63] Schulz KF, Grimes DA (2005). Sample size calculations in randomised trials: mandatory and mystical. Lancet.

[64] Department for Communities and Local Government (2015). English indices of deprivation 2015.

[65] Care Quality Commission. Community Mental Health Survey 2015. 2015. [Online]. Available at: http://www.cqc.org.uk/content/community-mental-health-survey-2015. Accessed 21 Oct 2015.

[66] Breslow NE, Clayton DG (1993). Approximate inference in generalized linear mixed models. J Am Stat Assoc.

[67] Tramm R, Daws K, Schadewaldt V (2013). Clinical trial recruitment—a complex intervention?. J Clin Nurs.

[68] Bagley HJ, Short H, Harman NL, Hickey HR, Gamble CL, Woolfall K, Young B, Williamson PR (2016). A patient and public involvement (PPI) toolkit for meaningful and flexible involvement in clinical trials—a work in progress. Res Involv Engagem.

[69] Stocks SJ, Giles SJ, Cheraghi-Sohi S, Campbell SM (2015). Application of a tool for the evaluation of public and patient involvement in research. BMJ Open.

[70] Coyne CA, Xu R, Raich P, Plomer K, Dignan M, Wenzel L, Fairclough D, Habermann T, Schnell L, Quella S, Cella D, E. C. O. Group (2003). Randomised, controlled trial of an easy-to-read informed consent statement for clinical trial participation: a study of the Eastern Cooperative Oncology Group. J Clin Oncol.

[71] Liénard J-L, Quinaux E, Fabre-Guillevin E, Piedbois P, Jouhaud A, Decoster G, Buyse M (2006). Impact of on-site initiation visits on patient recruitment and data quality in a randomized trial of adjuvant chemotherapy for breast cancer. Clin Trials.

[72] Kimmick GG, Peterson BL, Kornblith AB, Mandelblatt J, Johnson JL, Wheeler J, Heinze R, Cohen HJ, Muss HB (2005). Improving accrual of older persons to cancer treatment trials: a randomized trial comparing an educational intervention with standard information: CALGB 360001. J Clin Oncol.

[73] Hahn S, Puffer S, Torgerson DJ, Watson J (2005). Methodological bias in cluster randomised trials. BMC Med Res Methodol.

[74] Donner A, Klar N (2004). Pitfalls of and controversies in cluster randomization trials. Am J Public Health.

[75] Staley K, Buckland SA, Hayes H, Tarpey M (2014). ‘The missing links’: understanding how context and mechanism influence the impact of public involvement in research. Heal Expect.

[76] Buchbinder SP, Metch B, Holte SE, Scheer S, Coletti A, Vittinghoff E (2004). Determinants of Enrollment in a preventive HIV vaccine trial: hypothetical versus actual willingness and barriers to participation. JAIDS J Acquir Immune Defic Syndr.

[77] Wisdom K, Neighbors K, Williams VH, Havstad SL, Tilley BC (2002). Recruitment of African Americans with type 2 diabetes to a randomized controlled trial using three sources. Ethn Health.

[78] Graham AC, Raisch DW, Fye CL, Sather MR (2005). Assessment of the impact of a patient clinical trials handbook among pharmacy students. Clin Ther.

[79] Kruse AY, Kjaergard LL, Krogsgaard K, Gluud C, Mortensen EL, Gottschau A, Bjerg AM (2000). A randomised trial assessing the impact of written information on outpatients’ knowledge about attitude toward randomised clinical trials. The INFO trial group. Control Clin Trials.

[80] Snape D, Kirkham J, Britten N, Gradinger F, Looban F, Popay J (2014). Exploring perceived barriers, drivers, impacts and the need for evaluation of public involvement in health and social care research: a modified Delphi study. BMJ Open.

[81] Burns T, Catty J, Becker T, Drake RE, Fioritti A, Knapp M, Lauber C, Rössler W, Tomov T, van Busschbach J, White S, Wiersma D (2007). The effectiveness of supported employment for people with severe mental illness: a randomised controlled trial. Lancet.

[82] World Health Organization (2013). Mental Health Action Plan 2013–2020.

[83] World Health Organization (2013). Mental health: suicide prevention (SUPRE).

[84] Gamble C, Dudley L, Allam A, Bell P, Goodare H, Hanley B, Preston J, Walker A, Williamson P, Young B (2014). Patient and public involvement in the early stages of clinical trial development: a systematic cohort investigation. BMJ Open.

[85] Staley K. An evaluation of service user involvement in studies adopted by the Mental Health Research Network. MHRN. 2012. available at: http://www.twocanassociates.co.uk/perch/resources/files/MHRN%20Service_user_involvement_evaluation.pdf. Accessed 4 Dec 2016.

[86] Patterson S, Trite J, Weaver T (2014). Activity and views of service users involved in mental health research: UK survey. Br J Psychiatry.

[87] Larkey LK, Staten LK, Ritenbaugh C, Hall RA, Buller DB, Bassford T, Altimari BR (2002). Recruitment of Hispanic women to the Women’s Health Initiative. the case of Embajadoras in Arizona. Control Clin Trials.

[88] Ennis L, Wykes T (2013). Impact of patient involvement in mental health research: longitudinal study. Br J Psychiat.

[89] Free C, Hoile E, Robertson S, Knight R (2010). Three controlled trials of interventions to increase recruitment to a randomized controlled trial of mobile phone based smoking cessation support. Clin Trials.

[90] Corrigan PW, Watson AC (2002). The paradox of self-stigma and mental illness. Clin Psychol Sci Pract.

[91] Rüsch N, Angermeyer MC, Corrigan PW (2005). Mental illness stigma: concepts, consequences, and initiatives to reduce stigma. Eur Psychiatry.

[92] Clement S, Schauman O, Graham T, Maggioni F, Evans-Lacko S, Bezborodovs N, Morgan C, Rüsch N, Brown JSL, Thornicroft G (2014). What is the impact of mental health-related stigma on help-seeking? A systematic review of quantitative and qualitative studies. Psychol Med.

[93] Wilson P, Mathie E, Keenan J, McNeilly E, Goodman C, Howe A, Poland F, Staniszewska S, Kendall S, Munday D, Cowe M, Peckham S (2015). ReseArch with Patient and Public invOlvement: a RealisT evaluation – the RAPPORT study. Heal Serv Deliv Res.

[94] Knapp P, Raynor DK, Silcock J, Parkinson B (2009). Performance-based readability testing of participant materials for a phase I trial: TGN1412. J Med Ethics.

[95] Raynor DK, Knapp P, Silcock J, Parkinson B, Feeney K (2011). ‘User-testing’ as a method for testing the fitness-for-purpose of written medicine information. Patient Educ Couns.

[96] Durand M-A, Alam S, Grande SW, Elwyn G (2016). Much clearer with pictures’: using community-based participatory research to design and test a Picture Option Grid for underserved patients with breast cancer. BMJ Open.

[97] Edge D, Degnan A, Cotterill S, Berry K, Drake R, Baker R, Barrowclough C, Hughes-Morley A, Grey P, Bhugra D, Cahoon P, Tarrier N, Lewis S, Abel K (2016). Culturally-adapted Family Intervention (CaFI) for African-Caribbeans diagnosed with schizophrenia and their families: a feasibility study protocol of implementation and acceptability. Pilot Feasibility Stud.

[98] Moore GF, Audrey S, Barker M, Bond L, Bonell C, Hardeman W, Moore L, O’Cathain A, Tinati T, Wight D, Baird J (2015). Process evaluation of complex interventions: Medical Research Council guidance. BMJ..

