# Supplementary material for: The impact of advertising patient and public involvement on trial recruitment: embedded cluster randomised recruitment trial
Source: Trials. 2016 Dec 8;17:586. doi: 10.1186/s13063-016-1718-1 (PMC5146878; doi:10.1186/s13063-016-1718-1)
Supplement: Additional file 1: — Recruitment intervention advertising patient and public involvement in research. A copy of the recruitment intervention which was mailed to potential trial participants. (PDF 2112 kb) [file 13063_2016_1718_MOESM1_ESM.pdf]

Appendix 1: recruitment intervention advertising PPIR

# Take part in an award-winning study!

To find out more about the EQUIP study:


Telephone: 0161 306 7863

Email: equip@manchester.ac.uk


NHS
National Institute for Health Research

# Real patients and carers like you have helped to design this study

Outstanding Carer Involvement Award winning study

We are running a research study called EQUIP. Our study team includes real patients and carers, as well as researchers

**We hope you will consider taking part in the EQUIP study**

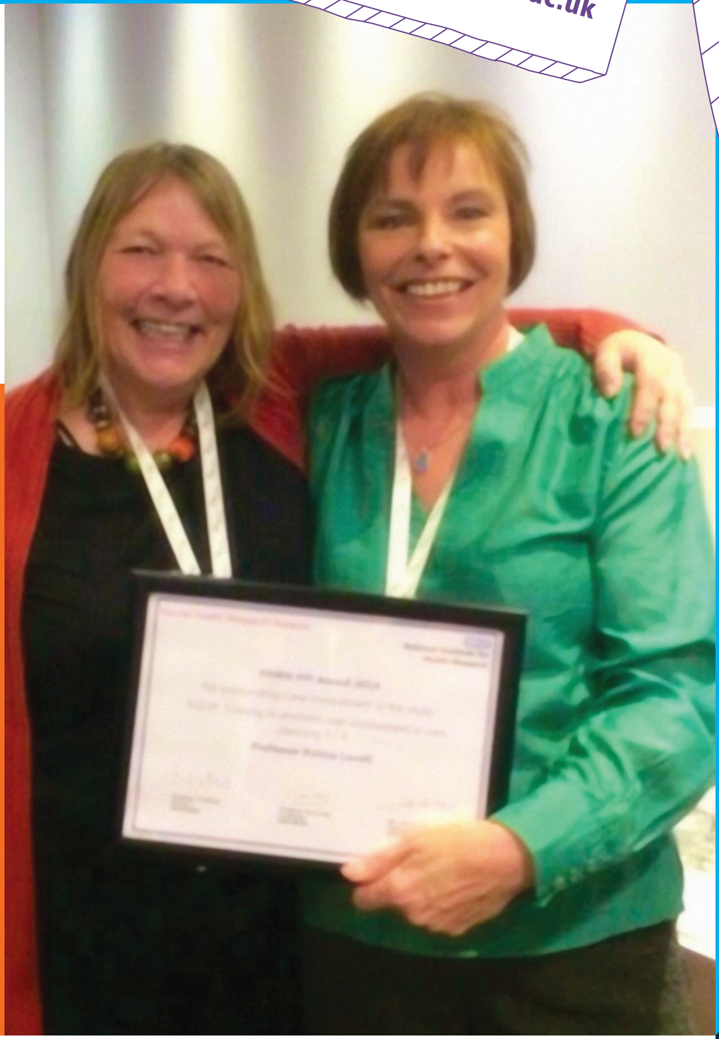

Professor Karina Lovell, EQUIP study lead, and Lindsey Cree, a carer and member of the study team, received the award from the Mental Health Research Network on behalf of the EQUIP study team.

> “I used to think research was just for academics, but since I’ve been involved in EQUIP I’ve realised how much my opinions matter and are taken on board”
>
> *Lindsey, Carer*

> “We have worked closely with patients and carers and are delighted that this has been recognised”
>
> *Professor Karina Lovell*

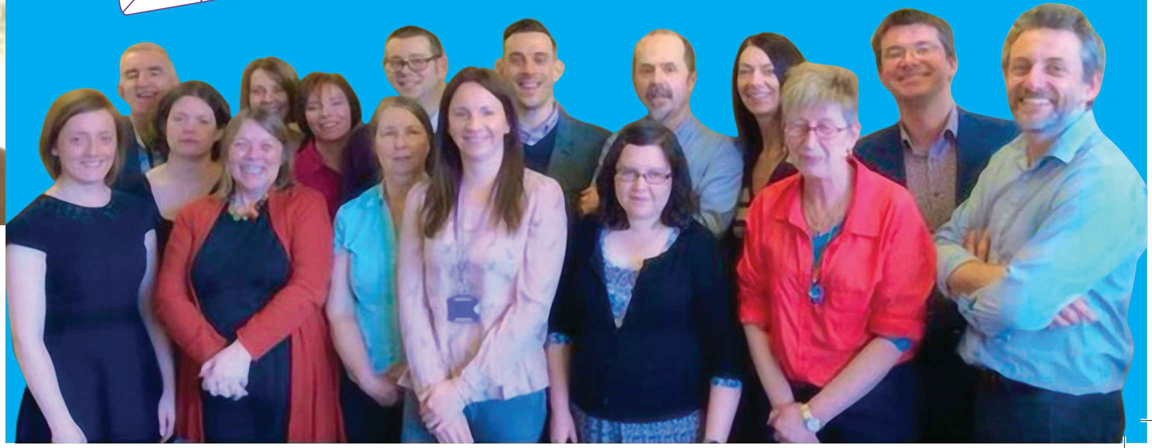

MANCHESTER 1824
The University of Manchester

EQUIP
Enhancing the quality of user involved care planning in mental health services


The EQUIP project is funded by the National Institute for Health Research’s Programme Grants for Applied Research Programme


MRC Hubs for Trials Methodology Research


This leaflet is a collaboration between the National Institute for Health Research Programme Grants for Applied Research (RP-PG-1210-12007) and Doctoral Research Fellowship (NIHRDRF-2012-05-1128)

# What do real patients and carers say about the EQUIP study?

**Donna is a patient.** As part of EQUIP she trains mental health professionals to better involve patients and carers in care planning.

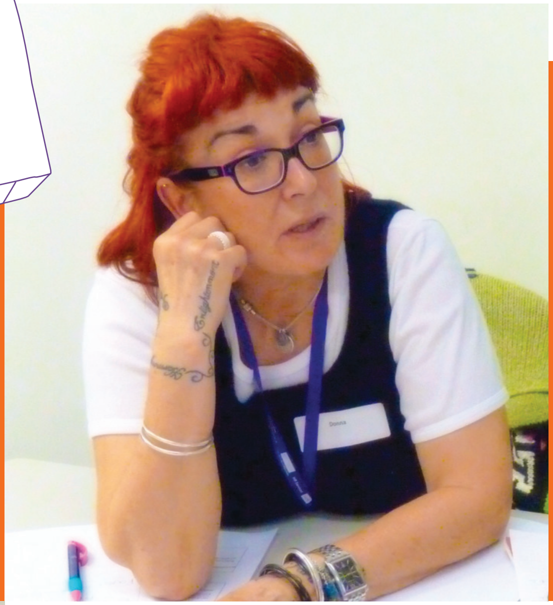

“As a patient, I think this study is very important because patients need to have more of a voice and involvement in the decisions about their own future well-being.”

*Donna, Patient*

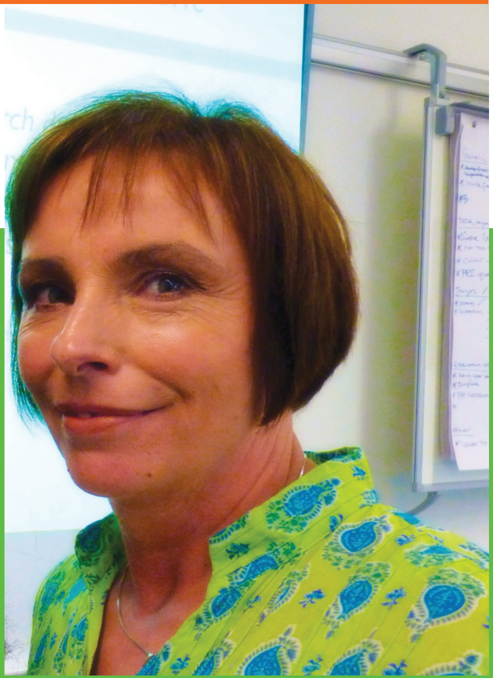

**Lindsey is a carer.** She helped to apply for funding for EQUIP and is part of the study management team. She also trains mental health professionals to better involve patients and carers in care planning as part of EQUIP.

“As a carer, I feel I am making a difference towards change in mental health care. If we don’t get involved in research, other people will be making decisions that can affect the future of people we care about. By taking part, I am making a difference to the future and there is no better feeling than that.”

*Lindsey, Carer*

# Real patients and carers say that by taking part in this study, you can help make a difference

Donna, a patient, and Lindsey, a carer, say their involvement in the EQUIP study is to focus on the needs of real patients and carers. They also helped to design this leaflet.

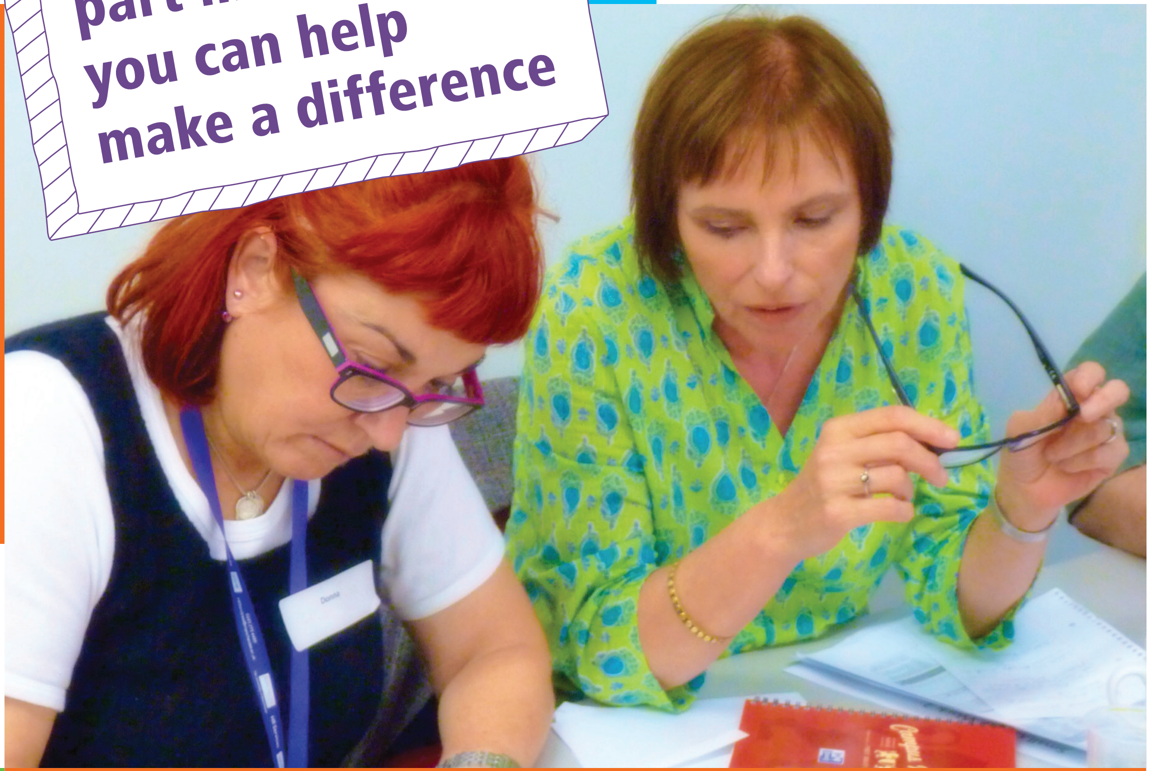

## Important things that we think you should remember:

- If you decide to take part in this study, you will be helping other patients in the future.
- The study appointments will be arranged at a time to suit you.
- You can pull out of the study at any time if you no longer want to take part, without giving a reason.
- EQUIP has been approved by an independent research ethics committee, to make sure patients taking part in it will not be harmed.
- We are asking you to consider taking part in this study because your care team think that you may be interested in taking part.
